# Human and mouse switch-like genes share common transcriptional regulatory mechanisms for bimodality

**DOI:** 10.1186/1471-2164-9-628

**Published:** 2008-12-23

**Authors:** Adam Ertel, Aydin Tozeren

**Affiliations:** 1 Center for Integrated Bioinformatics, School of Biomedical Engineering, Science and Health Systems, Drexel University, 3141 Chestnut Street, Philadelphia PA 19104 USA

## Abstract

**Background:**

Gene expression is controlled over a wide range at the transcript level through complex interplay between DNA and regulatory proteins, resulting in profiles of gene expression that can be represented as normal, graded, and bimodal (switch-like) distributions. We have previously performed genome-scale identification and annotation of genes with switch-like expression at the transcript level in mouse, using large microarray datasets for healthy tissue, in order to study the cellular pathways and regulatory mechanisms involving this class of genes. We showed that a large population of bimodal mouse genes encoding for cell membrane and extracellular matrix proteins is involved in communication pathways. This study expands on previous results by annotating human bimodal genes, investigating their correspondence to bimodality in mouse orthologs and exploring possible regulatory mechanisms that contribute to bimodality in gene expression in human and mouse.

**Results:**

Fourteen percent of the human genes on the HGU133A array (1847 out of 13076) were identified as bimodal or switch-like. More than 40% were found to have bimodal mouse orthologs. KEGG pathways enriched for bimodal genes included ECM-receptor interaction, focal adhesion, and tight junction, showing strong similarity to the results obtained in mouse. Tissue-specific modes of expression of bimodal genes among brain, heart, and skeletal muscle were common between human and mouse. Promoter analysis revealed a higher than average number of transcription start sites per gene within the set of bimodal genes. Moreover, the bimodal gene set had differentially methylated histones compared to the set of the remaining genes in the genome.

**Conclusion:**

The fact that bimodal genes were enriched within the cell membrane and extracellular environment make these genes as candidates for biomarkers for tissue specificity. The commonality of the important roles bimodal genes play in tissue differentiation in both the human and mouse indicates the potential value of mouse data in providing context for human tissue studies. The regulation motifs enriched in the bimodal gene set (TATA boxes, alternative promoters, methlyation) have known associations with complex diseases, such as cancer, providing further potential for the use of bimodal genes in studying the molecular basis of disease.

## Background

Our recent work applied an automated high-throughput method to classify genes with bimodal expression profiles within the mouse genome based on microarray experiments performed on healthy tissues using the Affymetrix MGU74Av2 microarray platform [1]. The identification of genes with bimodal expression is useful to identify the biological variation of genes that are tightly regulated around two discrete levels at the transcript level [2]. Many of the bimodal genes were expressed in "high" or "low" modes on a tissue-dependent basis. Enrichment analysis using Kyoto Encyclopedia of Genes and Genomes (KEGG) pathways [3] and Gene Ontology (GO) annotation [4] within this set of bimodal genes revealed that they are utilized in cell-cell communication and communication with the extracellular environment. We had also evaluated the expression of the bimodal genes in disease states for diabetes types I and II to reveal some of these genes with altered modes of expression in the disease state, revealing the roles of these genes in cell communication and immune response. As a natural extension of this work, we have applied the same automated high-throughput method to classify genes with bimodal expression in the human genome and compared the list with human orthologs of mouse bimodal genes. Moreover, we looked into the transcript-level regulation of bimodal genes using a variety of bioinformatics databases.

The detection of bimodal genes in human is useful for determining a set of genes tightly controlled around two states at the transcript level. Additionally, the identification of these bimodal genes provides an indication of how well the previous methods extend across species and different microarray platforms. While it is expected that many orthologs between human and mouse would share patterns of regulation such as bimodality, the literature has also documented that many gene regulation promoters have changed over the course of evolution between human and mouse [5]. Genes with bimodal expression profiles in both organisms may indicate conservation of alternative promoters, which have been implicated in tissue-specific expression common among the bimodal genes. Alternately, genes with known orthologs that have been identified as bimodal in only one of these species, may illustrate the instability in mammalian promoters [5,6]. Investigation of the regulatory mechanisms at play in the expression of bimodal genes should provide insight into the stability of their expression as well as how these genes may lose regulation in the process of disease [7].

There are many factors contributing to the regulation of transcription, having varying impact in the difference in expression level and the time scale over which the expression level may change, either within a cell or over a course of cell divisions. Transcription factors may enhance or inhibit expression as they bind to regulatory gene promoters effecting transcription initiation [8]. Changes in transcription factor activity may account for bimodality in genes within a single tissue over time, such as in circadian rhythm pathways [9]. Transcript-level regulation may also be achieved through epigenetic modification, such as CpG island methylation, which inhibits transcription either at the promoter region or downstream [10,11]. Additional epigenetic mechanisms, including histone modifications such as methylation and acetylation were shown to be associated with transcription initiation and elongation [12]. These epigenetic regulatory mechanisms may be linked with bimodality resulting from differentiated tissues, where stable modifications maintain a high mode of expression in a certain number of tissues, and a low mode of expression in others. Regulatory mechanisms mentioned above may work in combination to produce a variety of expression profiles, where even in bimodal genes one mechanism may account for expression level changes within a single mode of expression and an alternative regulatory mechanism may account for expression level changes between modes of expression.

In this study, we extend our classification of bimodal genes to the human genome. Our results indicate that a sizable number of genes with bimodal expression in mouse are also bimodal in human, with recurring roles of cell-cell communication and communication with the extracellular environment. Furthermore, the set of bimodal genes identified by our method shows a strong connection to epigenetic regulation, namely methylation of histone tails in gene promoter regions.

## Results

### Identification of bimodal genes in the human genome

Microarray data for tissue types listed in table 1 (See Additional file 1 for dataset and sample accession numbers) were used to identify bimodal genes in the human genome. Two-component mixture analysis of the 13076 unique genes represented in both Affymetrix HGU133A and HGU133plus2 microarrays identified 1847 genes, or 14%, as bimodal, with p < 0.001. Among these genes with orthologs in mouse, 42% were identified in our previous study on MGU74Av2 microarrays. The probability of obtaining this overlap from a random selection of genes, estimated from the hypergeometric distribution, is indistinguishable from zero. Additional file 2 provides the list of bimodal genes accompanied by the mouse ortholog, as well as the p-value and threshold between high and low modes of expression, *X*_T_.

**Table 1:** Microarray datasets used in this study representing normal human tissue.

Tissue	Samples	(Used in mouse study)
adipose	10	(6)
adrenal	10	(6)
brain	89	(89)
colon	10	(5)
epidermal	25	(25)
heart	38	(38)
kidney	10	(3)
liver	10	(8)
lung	26	(26)
mammary	15	(15)
muscle	64	(64)
ovary	10	(10)
pancreas	6	(5)
peripheral_blood	12	(12)
small_intestine	7	(3)
spleen	12	(12)
stomach	10	(1)
testis	38	(49)
thymus	5	(11)

total	407	(388)

The number of tissue samples used in the previous work that identified bimodal genes in mouse are included in parentheses. Sample accession numbers from GEO and ArrayExpress are listed in Additional File 1.

### Functional enrichment analysis highlights themes of cell communication

**Figure 1:**
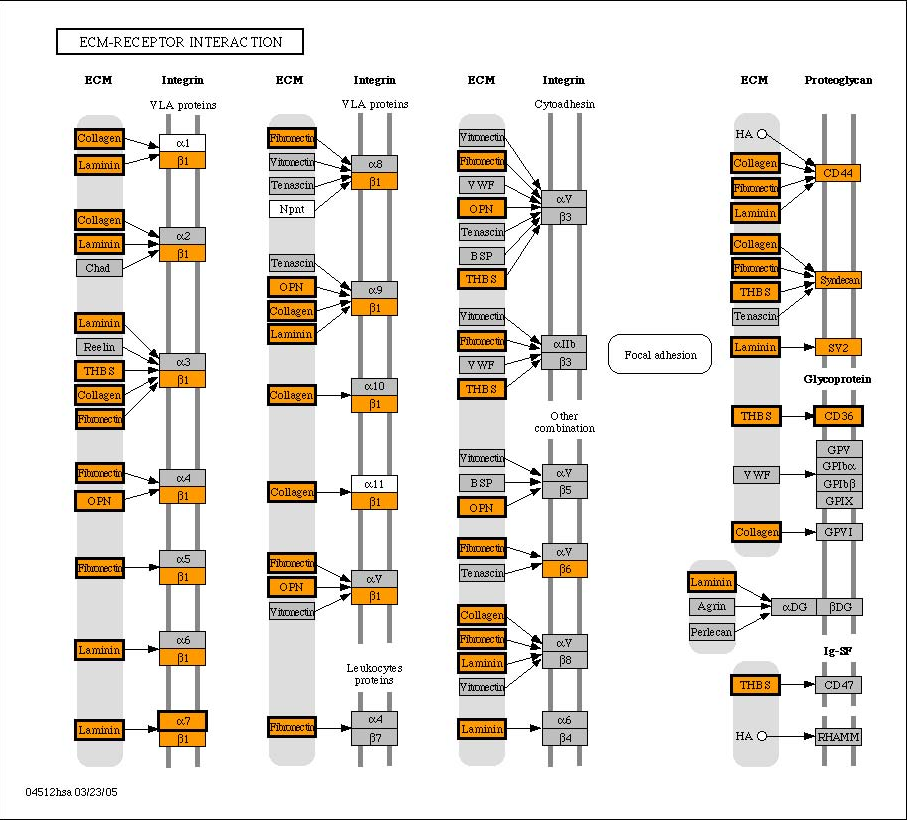
**ECM-receptor interaction pathway enriched by human switch-like genes**. Nodes enriched for human bimodal genes are colored orange, while nodes also identified as bimodal in mouse orthologs are outlined in bold. In all, the overlap between bimodal human and bimodal mouse orthologs contains thirteen unique genes represented in seven unique nodes in the ECM-receptor pathway. Nodes colored in gray were not identified as bimodal, while white nodes are used for genes that are not represented on the HGU133A array.

**Figure 2:**
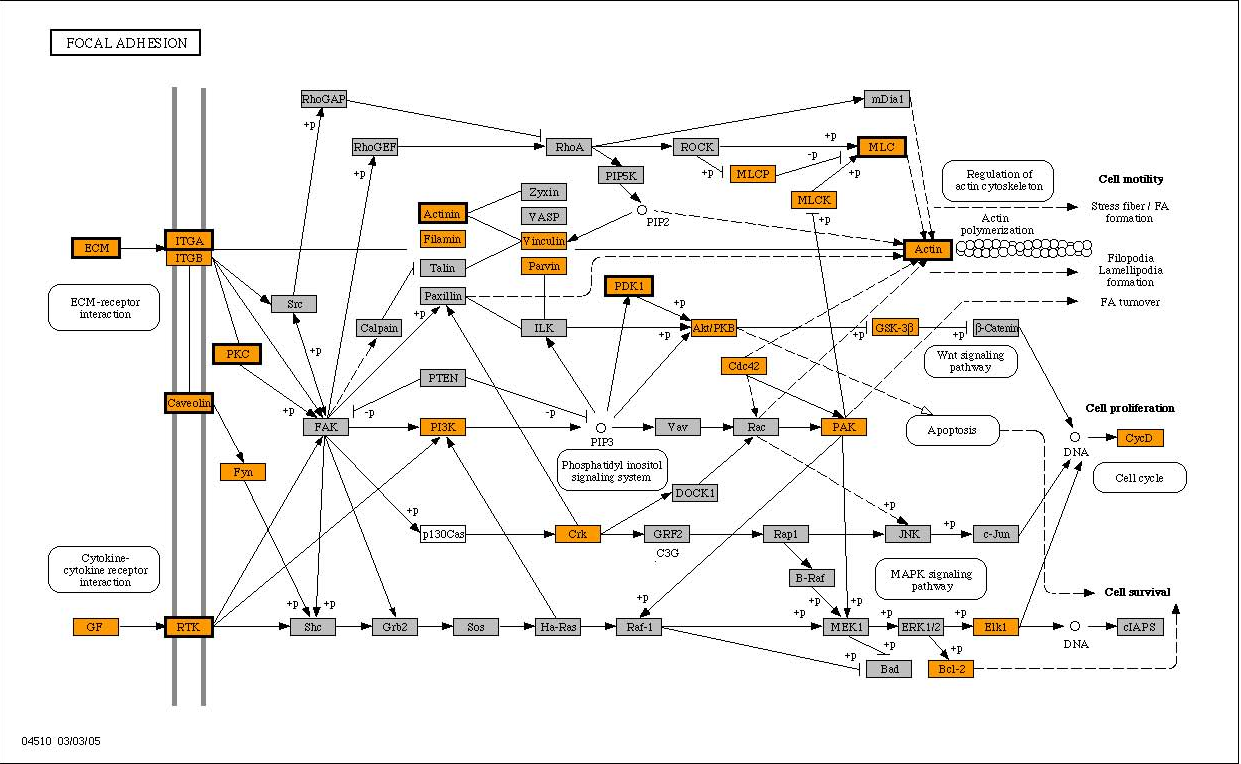
**KEGG Focal adhesion pathway enriched by human switch-like genes**. Nodes enriched for human bimodal genes are colored orange, while nodes also identified as bimodal in mouse orthologs are outlined in bold. In all, the overlap between human and mouse orthologs contains twenty-two unique genes represented in nine unique nodes in the focal adhesion pathway. Nodes colored in gray were not identified as bimodal, while white nodes are used for genes that are not represented on the HGU133A array.

Enrichment of KEGG pathways and GO terms extended the theme of communication with neighboring cells and the extracellular environment that was also evident from bimodal genes in mouse. Enriched KEGG pathways are presented in Table 2, while enriched GO terms are detailed in Table 3, including listings for cellular component, biological process, and molecular function terms. A majority of the KEGG pathways previously identified as enriched for bimodal genes in mouse were also significantly enriched in humans (Table 2). The most highly populated pathways common to bimodal human genes and human orthologs for bimodal mouse genes are the calcium signaling, focal adhesion and tight junction pathways. The cell communication pathways ECM-receptor interaction and focal adhesion, which were identified as significant within the last study, again appeared as highly populated with bimodal genes. The KEGG ECM-receptor interaction pathway is shown in Figure 1, with enriched nodes highlighted in orange, and nodes replicated in the mouse study outlined in bold. The figure shows that integrin subunits a7, B1, and B6 and a subset of their receptors including multiple collagen types I, II, and IV, fibronectin and laminin are bimodal, as would be expected since these proteins contribute to tissue specificity. Also identified bimodal are the cell membrane receptors CD44, SV2, CD36, and Syndecan. The KEGG focal adhesion pathway, which also interacts with the ECM, is depicted in Figure 2. The figure shows that the bimodal genes are not only positioned at ECM and cell membrane but also at different stages of cell signaling, indicating the extensive role that bimodal genes play in the processing of crosstalk between cells and the ECM. Proteins that are deemed as bimodal in this pathway include EGF, ELK1, FYN, HGF, vinculin, actinin and cyclins D1 and D2. Additional file 3 presents the list of bimodal genes along the enriched KEGG pathways. Further enrichment analysis for the sets of bimodal genes expressed in the "high" and "low" modes was performed on three tissue types with the most abundant samples. Enrichment of the "high" and "low" mode subsets revealed tissue-specific activation and deactivation, as may be expected for pathways and GO terms describing specialized functions such as muscle contraction or synaptic transmission. The KEGG pathways listed in Table 4 and the GO terms listed in Table 5 demonstrate the tissue-specific activation and deactivation of bimodal genes and show consistency between human and mouse for several terms.

**Table 2:** KEGG pathway enrichment for human switch-like genes.

	Human bimodal genes				Mouse bimodal genes			
KEGG Pathway	Genes Observed	Genes Expected	Ration of Enrichment	P-values	Genes Observed	Genes Expected	Ration of Enrichment	P-values
**Tight junction**	**39**	**17.09**	**2.28**	**2.87E-07**	**30**	**14.60**	**2.05**	**4.52E-05**
Cell adhesion molecules (CAMs)	36	16.81	2.14	4.31E-06	*23*	*16.92*	*1.36*	*7.17E-02*
Regulation of actin cytoskeleton	51	27.54	1.85	6.10E-06	*27*	*25.38*	*1.06*	*3.95E-01*
**Focal adhesion**	**50**	**26.84**	**1.86**	**6.22E-06**	**43**	**23.89**	**1.80**	**4.77E-05**
Calcium signaling pathway	44	23.31	1.89	1.54E-05	*25*	*20.90*	*1.20*	*1.91E-01*
Citrate cycle (TCA cycle)	11	3.53	3.12	2.85E-04	*6*	*3.98*	*1.51*	*1.96E-01*
Glycolysis/Gluconeogenesis	18	7.91	2.28	4.64E-04	*14*	*7.47*	*1.88*	*1.16E-02*
**PPAR signaling pathway**	**19**	**8.62**	**2.21**	**5.10E-04**	**17**	**9.46**	**1.80**	**9.09E-03**
Long-term potentiation	20	9.32	2.15	5.47E-04	*15*	*8.13*	*1.85*	*1.08E-02*
Leukocyte transendothelial migration	28	15.11	1.85	7.12E-04	*20*	*14.10*	*1.42*	*6.15E-02*
Carbon fixation	9	2.97	3.03	1.29E-03	*7*	*2.99*	*2.34*	*2.00E-02*
Adherens junction	20	10.17	1.97	1.81E-03	*13*	*10.12*	*1.28*	*2.02E-01*
Gap junction	24	13.14	1.83	2.03E-03	*16*	*10.95*	*1.46*	*7.02E-02*
Melanogenesis	23	12.57	1.83	2.44E-03	*14*	*12.94*	*1.08*	*4.19E-01*
Type I diabetes mellitus	13	5.65	2.30	2.49E-03	*11*	*7.80*	*1.41*	*1.44E-01*
Alanine and aspartate metabolism	10	3.96	2.53	3.57E-03	*6*	*3.65*	*1.64*	*1.44E-01*
Neurodegenerative Diseases	12	5.23	2.30	3.67E-03	*5*	*4.98*	*1.00*	*5.72E-01*
MAPK signaling pathway	50	34.61	1.44	4.14E-03	*36*	*31.36*	*1.15*	*2.04E-01*
Reductive carboxylate cycle (CO2 fixation)	5	1.27	3.93	4.29E-03	*2*	*1.66*	*1.21*	*5.13E-01*
Endometrial cancer	14	7.06	1.98	7.76E-03	*11*	*6.80*	*1.62*	*6.59E-02*
Pyruvate metabolism	11	5.09	2.16	8.75E-03	*7*	*4.81*	*1.45*	*1.94E-01*
**ECM-receptor interaction**	**20**	**11.58**	**1.73**	**8.98E-03**	**25**	**10.45**	**2.39**	**1.00E-05**
Long-term depression	*18*	*10.45*	*1.72*	*1.31E-02*	17	9.12	1.86	6.16E-03
Cell Communication	*25*	*16.10*	*1.55*	*1.53E-02*	34	15.43	2.20	2.47E-06
Insulin signaling pathway	*27*	*18.36*	*1.47*	*2.38E-02*	26	15.93	1.63	6.19E-03
Fructose and mannose metabolism	*7*	*4.94*	*1.42*	*2.17E-01*	12	5.31	2.26	3.63E-03
Cysteine metabolism	*2*	*2.12*	*0.94*	*6.47E-01*	4	1.00	4.02	8.53E-03

KEGG pathways enrichment was computed using the set of human bimodal genes as well as previously identified bimodal genes in mouse. Pathways enriched with p ≤ 0.01 in both bimodal human genes and the set of bimodal mouse genes are shown in bold. Italicized values do not meet the p ≤ 0.01 significance threshold.

**Table 3:** Gene ontology enrichment for human switch-like genes.

		Human bimodal genes				Mouse bimodal genes			
Gene Ontology		Genes Observed	Genes Expected	Ration of Enrichment	P-values	Genes Observed	Genes Expected	Ration of Enrichment	P-values
CC	**Cytoplasm**	**527**	**408.92**	**1.29**	**0.00E+00**	**395**	**335.13**	**1.18**	**3.73E-05**
	**cytoskeleton**	**110**	**57.49**	**1.91**	**0.00E+00**	**68**	**41.97**	**1.62**	**1.91E-05**
	**sarcoplasmic reticulum**	**14**	**2.40**	**5.83**	**5.39E-10**	**10**	**2.49**	**4.02**	**2.06E-05**
	**membrane**	**680**	**577.01**	**1.18**	**2.03E-08**	**494**	**442.64**	**1.12**	**9.12E-04**
	striated muscle thick filament	12	2.26	5.31	6.39E-08	*7*	*1.99*	*3.52*	*1.25E-03*
	muscle myosin complex	12	2.54	4.72	4.94E-07	*0*	*N/A*	*N/A*	*N/A*
	actin cytoskeleton	36	15.68	2.30	6.87E-07	*11*	*8.13*	*1.35*	*1.78E-01*
	myosin complex	21	6.92	3.03	9.19E-07	*13*	*5.81*	*2.24*	*2.76E-03*
	neuromuscular junction	6	0.85	7.08	7.89E-06	*6*	*2.65*	*2.26*	*3.69E-02*
	**cell junction**	**62**	**37.01**	**1.68**	**2.09E-05**	**55**	**28.20**	**1.95**	**2.64E-07**
	Z disc	9	1.98	4.55	2.24E-05	*8*	*3.48*	*2.30*	*1.51E-02*
	**synapse**	**41**	**22.04**	**1.86**	**4.26E-05**	**41**	**20.07**	**2.04**	**2.33E-06**
	membrane fraction	97	66.25	1.46	5.02E-05	*32*	*27.71*	*1.15*	*2.10E-01*
	growth cone	10	2.68	3.73	8.41E-05	*9*	*3.32*	*2.71*	*2.72E-03*
	plasma membrane	239	192.10	1.24	9.90E-05	*169*	*143.67*	*1.18*	*9.51E-03*
	cell projection	23	11.02	2.09	3.38E-04	*19*	*10.62*	*1.79*	*6.35E-03*
	costamere	4	0.57	7.08	3.97E-04	*1*	*0.66*	*1.51*	*5.16E-01*
	clathrin coat of trans-Golgi network vesicle	6	1.27	4.72	4.51E-04	*0*	*N/A*	*N/A*	*N/A*
	sarcoplasmic reticulum membrane	6	1.27	4.72	4.51E-04	*5*	*1.16*	*4.31*	*1.95E-03*
	**proteinaceous extracellular matrix**	**52**	**33.62**	**1.55**	**7.38E-04**	**60**	**28.04**	**2.14**	**1.47E-09**
	integral to plasma membrane	184	148.88	1.24	9.15E-04	*53*	*47.62*	*1.11*	*2.14E-01*
	**troponin complex**	**5**	**0.99**	**5.06**	**9.16E-04**	**6**	**1.33**	**4.52**	**4.27E-04**
	axon	*10*	*3.81*	*2.62*	*2.62E-03*	18	7.96	2.26	3.97E-04
	sarcolemma	*6*	*1.70*	*3.54*	*3.39E-03*	13	3.82	3.41	1.51E-05
	basement membrane	*12*	*5.79*	*2.07*	*9.14E-03*	17	5.81	2.93	1.12E-05
	collagen	*7*	*2.83*	*2.48*	*1.60E-02*	14	4.81	2.91	7.34E-05
	postsynaptic membrane	*19*	*12.71*	*1.49*	*4.48E-02*	21	10.29	2.04	6.69E-04
	I band	*2*	*0.42*	*4.72*	*5.42E-02*	4	0.66	6.03	7.55E-04
	synaptic vesicle membrane	*1*	*0.28*	*3.54*	*2.63E-01*	6	1.33	4.52	4.27E-04
	basal lamina	*2*	*1.13*	*1.77*	*3.15E-01*	9	2.49	3.62	1.79E-04

BP	**muscle contraction**	**39**	**12.43**	**3.14**	**0.00E+00**	**10**	**2.82**	**3.55**	**9.66E-05**
	**striated muscle contraction**	**21**	**4.10**	**5.13**	**0.00E+00**	**11**	**3.15**	**3.49**	**5.21E-05**
	**cell adhesion**	**112**	**65.68**	**1.71**	**3.76E-09**	**81**	**44.63**	**1.81**	**1.67E-08**
	nervous system development	79	42.38	1.86	1.36E-08	*33*	*22.40*	*1.47*	*1.19E-02*
	**muscle development**	**29**	**10.03**	**2.89**	**2.95E-08**	**14**	**5.31**	**2.64**	**2.72E-04**
	sensory perception of sound	33	15.26	2.16	8.44E-06	*5*	*4.98*	*1.00*	*5.72E-01*
	glycolysis	19	7.06	2.69	2.44E-05	*15*	*6.97*	*2.15*	*2.11E-03*
	regulation of heart contraction	13	3.96	3.29	4.01E-05	*9*	*3.48*	*2.58*	*4.07E-03*
	**cytoskeleton organization and biogenesis**	**24**	**10.74**	**2.24**	**7.75E-05**	**26**	**13.11**	**1.98**	**2.72E-04**
	antigen processing and presentation of peptide antigen via MHC class I	7	1.41	4.96	8.99E-05	*6*	*2.49*	*2.41*	*2.67E-02*
	dephosphorylation	24	10.88	2.21	9.77E-05	*11*	*9.12*	*1.21*	*2.98E-01*
	**regulation of muscle contraction**	**8**	**1.84**	**4.36**	**1.03E-04**	**13**	**2.82**	**4.61**	**8.48E-08**
	regulation of striated muscle contraction	6	1.13	5.31	1.71E-04	*2*	*0.50*	*4.02*	*7.34E-02*
	central nervous system development	25	12.01	2.08	1.99E-04	*5*	*4.81*	*1.04*	*5.39E-01*
	cell differentiation	72	47.88	1.50	1.99E-04	*54*	*49.44*	*1.09*	*2.57E-01*
	multicellular organismal development	136	102.41	1.33	2.26E-04	*84*	*85.11*	*0.99*	*5.73E-01*
	regulation of the force of heart contraction	5	0.85	5.90	2.96E-04	*4*	*1.66*	*2.41*	*6.86E-02*
	neuromuscular synaptic transmission	5	0.85	5.90	2.96E-04	*3*	*1.16*	*2.58*	*9.46E-02*
	neuron migration	12	4.10	2.93	3.05E-04	*8*	*8.63*	*0.93*	*6.50E-01*
	neuron differentiation	13	4.66	2.79	3.08E-04	*4*	*5.81*	*0.69*	*8.55E-01*
	glycogen metabolic process	12	4.24	2.83	4.44E-04	*3*	*3.32*	*0.90*	*6.68E-01*
	protein amino acid dephosphorylation	29	15.54	1.87	5.06E-04	*11*	*11.95*	*0.92*	*6.67E-01*
	tricarboxylic acid cycle	10	3.39	2.95	9.10E-04	*6*	*3.65*	*1.64*	*1.44E-01*
	very-long-chain fatty acid metabolic process	5	0.99	5.06	9.16E-04	*1*	*0.50*	*2.01*	*4.20E-01*
	ion transport	*83*	*62.01*	*1.34*	*2.89E-03*	67	44.13	1.52	1.88E-04
	transport	*218*	*188.15*	*1.16*	*8.19E-03*	188	153.46	1.23	9.54E-04
	calcium ion transport	*20*	*11.72*	*1.71*	*1.03E-02*	21	8.96	2.34	7.22E-05
	synaptic transmission	*35*	*24.01*	*1.46*	*1.28E-02*	21	7.96	2.64	8.49E-06

MF	actin binding	78	34.32	2.27	0.00E+00	*42*	*27.04*	*1.55*	*1.76E-03*
	structural constituent of muscle	28	5.65	4.96	0.00E+00	*5*	*1.49*	*3.35*	*8.74E-03*
	**structural molecule activity**	**81**	**42.52**	**1.91**	**3.01E-09**	**58**	**34.01**	**1.71**	**1.53E-05**
	structural constituent of cytoskeleton	32	12.15	2.63	8.12E-08	*22*	*11.45*	*1.92*	*1.26E-03*
	**protein binding**	**777**	**677.86**	**1.15**	**1.68E-07**	**558**	**497.39**	**1.12**	**1.79E-04**
	calmodulin binding	37	16.95	2.18	1.93E-06	*23*	*13.11*	*1.75*	*3.73E-03*
	**motor activity**	**33**	**14.69**	**2.25**	**3.40E-06**	**28**	**13.60**	**2.06**	**7.77E-05**
	**calcium ion binding**	**145**	**103.40**	**1.40**	**8.50E-06**	**116**	**76.98**	**1.51**	**1.37E-06**
	GTPase activity	43	24.15	1.78	8.61E-05	*22*	*19.74*	*1.11*	*3.24E-01*
	phosphoprotein phosphatase activity	31	15.96	1.94	1.52E-04	*10*	*13.44*	*0.74*	*8.85E-01*
	phosphoric monoester hydrolase activity	25	11.87	2.11	1.61E-04	*12*	*10.12*	*1.19*	*3.07E-01*
	microfilament motor activity	9	2.40	3.75	1.82E-04	*3*	*1.00*	*3.01*	*6.15E-02*
	NAD binding	13	4.52	2.88	2.14E-04	*3*	*4.48*	*0.67*	*8.49E-01*
	transferase activity, transferring phosphorus-containing groups	7	1.55	4.51	2.17E-04	*6*	*1.66*	*3.62*	*2.36E-03*
	protein tyrosine phosphatase activity	25	12.43	2.01	3.59E-04	*14*	*10.62*	*1.32*	*1.65E-01*
	transporter activity	61	40.68	1.50	6.35E-04	*41*	*33.18*	*1.24*	*8.24E-02*
	long-chain-fatty-acid-CoA ligase activity	6	1.41	4.25	9.92E-04	*3*	*1.33*	*2.26*	*1.33E-01*
	creatine kinase activity	*3*	*0.57*	*5.31*	*1.01E-02*	4	0.66	6.03	7.55E-04
	extracellular matrix structural constituent	*18*	*10.88*	*1.65*	*1.96E-02*	18	6.80	2.65	3.52E-05
	ion channel activity	*48*	*36.02*	*1.33*	*2.15E-02*	46	25.38	1.81	2.17E-05

GO category enrichment was computed using the set of human bimodal genes as well as the previously identified bimodal genes in mouse. GO terms enriched with p ≤ 0.001 in both bimodal human genes and the set of bimodal mouse genes are shown in bold. Italicized values do not meet the p ≤ 0.001 significance threshold

**Table 4:** KEGG pathways enriched for bimodal genes with "high" or "low" expression within brain, heart, and skeletal muscle tissues in human and mouse.

	Brain		Heart		Skeletal Muscle	
	Human	Mouse	Human	Mouse	Human	Mouse
Calcium signaling pathway					high	
Carbon fixation			high	High	high	
Cell Communication	low	low	high	High		high
Cell adhesion molecules (CAMs)					low	
Citrate cycle (TCA cycle)				high	high	
ECM-receptor interaction	low	low	high	high	high	high
Focal adhesion	low	low	high	high	high	high
Gap junction	high	high		low	low	low
Glycolysis/Gluconeogenesis			high	high	high	high
Insulin signaling pathway				high		
Leukocyte transendothelial migration						high
Long-term depression	high		low	low	low	low
Long-term potentiation		high	low			
Neurodegenerative Diseases	high					
Pyruvate metabolism			high	high	high	
Reductive carboxylate cycle (CO2 fixation)			high		high	
Regulation of actin cytoskeleton						high
Type I diabetes mellitus					low	

The mode of expression is indicated for tissue types where pathways are significantly enriched for the set of bimodal genes in the "high" mode or the set of bimodal genes in the "low" mode of expression. Significance was determined from a hypergeometric distribution with cutoff p = 0.05.

**Table 5:** Gene Ontology terms enriched for bimodal genes with "high" or "low" expression within brain, heart, and skeletal muscle tissues in human and mouse.

		Brain		Heart		Skeletal Muscle	
		Human	Mouse	Human	Mouse	Human	Mouse
CC	actin cytoskeleton			high		high	high
	axon	high	high	low	low	low	low
	basal lamina		low				
	basement membrane		low		high		high
	cell junction	high	high		low	low	low
	cell projection	high	high				
	collagen	low	low		high	high	high
	cytoplasm	high		high		high	
	cytoskeleton		low	high	high	high	high
	growth cone	high	high	low			
	I band						high
	integral to plasma membrane					low	
	membrane		high			low	
	membrane fraction				low		low
	muscle myosin complex	low				high	
	myosin complex	low	low				high
	plasma membrane					low	
	postsynaptic membrane	high	high	low	low		low
	proteinaceous extracellular matrix	low	low	high	high		high
	sarcolemma		low		high		high
	sarcoplasmic reticulum	low	low	high		high	high
	striated muscle thick filament	low				high	
	synapse	high	high	low	low	low	low
	synaptic vesicle membrane						low
	troponin complex		low			high	high
	Z disc	low	low	high	high	high	high

BP	antigen processing and presentation of peptide antigen via MHC class I		low	high			high
	cell adhesion		low	high	high		high
	cell differentiation		high				
	central nervous system development	high		low			
	cytoskeleton organization and biogenesis						high
	glycogen metabolic process	low			high	high	
	glycolysis			high	high	high	
	ion transport	high	high		low		low
	muscle contraction	low	low	high		high	high
	muscle development	low				high	high
	nervous system development	high	high	low		low	
	neuron differentiation		high	low			
	neuron migration		high				
	regulation of heart contraction			high	high		
	regulation of muscle contraction		low				high
	regulation of striated muscle contraction					high	
	regulation of the force of heart contraction			high	high	high	
	sensory perception of sound		high	high			
	striated muscle contraction	low	low	high		high	
	synaptic transmission	high	high	low	low	low	low
	transport	high	high		low		low
	tricarboxylic acid cycle			high	high	high	high

MF	actin binding			high		high	high
	calcium ion binding			high		high	high
	extracellular matrix structural constituent	low	low	high	high		high
	GTPase activity	high					
	ion channel activity		high		low		low
	microfilament motor activity					high	
	NAD binding					high	
	protein binding	low					
	structural constituent of muscle	low	low	high		high	
	structural molecule activity		low	high			

The mode of expression is indicated for tissue types where terms are significantly enriched for the set of bimodal genes in the "high" mode or the set of bimodal genes in the "low" mode of expression. Significance was determined from a hypergeometric distribution with cutoff p = 0.05.

### Promoter analysis reveals bias for TATA boxes in bimodal genes

**Figure 3:**
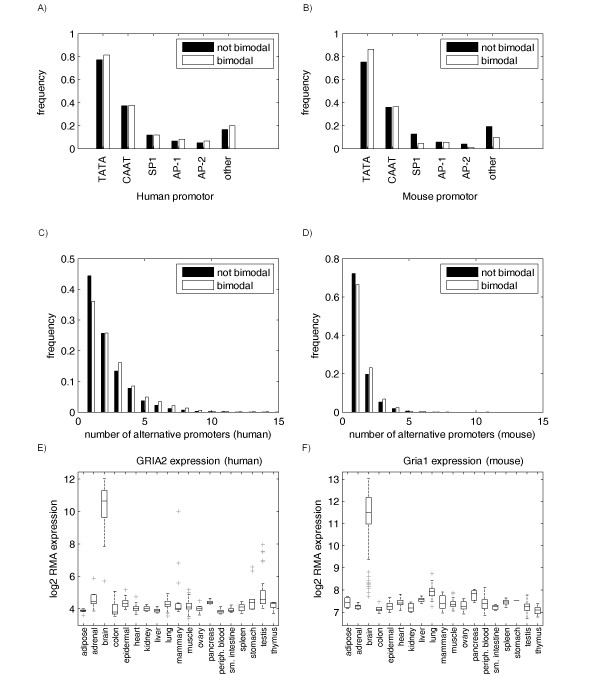
**Summary of promoter usage between bimodal and non-bimodal subsets**. The relative frequency of core promoter types cataloged in MPromDB [13] is shown for bimodal and non-bimodal gene subsets in A) human and B) mouse. The number of alternative promoters per gene is shown for bimodal genes and non-bimodal genes for C) human and D) mouse. For a subset of bimodal genes with multiple alternative promoters, tissue-dependent alternative promoters from DBTSS [14] corresponded to the mode of expression, as shown for glutamate receptors E) GRIA2 in human and F) Gria1 in mouse.

The mammalian promoter database (MPromDB) [13] was used to assess the distribution of common promoter types within the set of bimodal genes. MPromDB contained promoters for 840 genes represented on the HGU133 arrays and promoters for 536 genes represented on the MGU74Av2 array. The frequencies of the common promoter types AP-1, AP-2, SP1, TATA and CAAT are illustrated in Figure 3A and 3B for the sets of bimodal and non-bimodal genes in human and mouse. The remaining promoter types seldom appeared and were bundled together into an "other" category. The set of bimodal genes within human and mouse shows a statistically significant bias for TATA promoters, with significance of p = 9.5e-5 for human and p = 4.9e-7 for mouse, estimated from a hypergeometric distribution. The remaining promoter types present between bimodal and non-bimodal genes revealed no significant differences that were consistent between human and mouse, suggesting that bimodality in gene expression is largely independent of the regulatory promoter type. The SP1 and AP1 promoters appeared underrepresented in mouse bimodal genes, but this result is based on only a small subset of genes and was not consistent with the results in human.

### Alternative promoter sites more common in bimodal genes

The database of transcription start sites (DBTSS) [14] was used to evaluate the number of alternative promoters associated with genes in the bimodal and non-bimodal subsets for human and mouse. The distribution of alternative promoters was shifted towards a higher number of promoters per gene for those with bimodal distributions, as shown in Figure 3C and 3D, providing evidence for some contribution towards the dynamic range of gene expression required for bimodality. When compared against non-bimodal genes, two or more promoters are more common in bimodal genes for both human and mouse, with a respective significance level of p ≈ 0 and p = 1.9e-8, estimated from a hypergeometric distribution. Multiple promoters per gene may be prevalent but not required for bimodal expression. Alternative promoter sites in human and mouse were tested for tissue-selective usage corresponding to the mode of expression for bimodal genes. Alternative promoters for 168 bimodal genes in human were identified as corresponding with the mode of expression across within the nineteen tissues. Alternative promoter data were not available for skeletal muscle tissue in mouse, but data for the remaining 18 tissues identified 131 genes with at least one alternative promoter corresponding to the mode of expression. Random permutation of the tissue labels was used to estimate a median false discovery rate of 4%. Though there was no overlap between these tissue-selective promoter gene sets in human and mouse, there were several pathways in common for this comparison, including the neuroactive ligand-receptor interaction, gap junction, and calcium signaling pathway. The alternative promoters identified as corresponding to the mode of expression in both human and mouse were largely brain-specific. For example, the genes GRIA2 in human and Gria1 in mouse encoding for glutamate receptor proteins in the neuroactive ligand-receptor interaction pathway were associated with multiple alternative promoters specific to brain. Expression box plots across the nineteen tissues having at least one promoter specific to brain, is shown in Figure 3E and 3F for human GRIA2 and mouse Gria1 genes, respectively. These results indicate that multiple alternative promoters may provide redundancy and that a single mode of expression does not necessarily correspond with a unique alternative promoter.

### DNA methylation shows a negligible contribution to bimodal gene expression

Cytosine methylation has been shown to provide a stable mechanism in mammals for altering DNA-protein interactions [10]. Genes can be transcribed from methylation-free promoters even when adjacent transcribed and nontranscribed regions are extensively methylated [10]. Methylation of CpG-rich promoters prevents transcriptional initiation and ensures the silencing of imprinted genes and genes in the X Chromosome [10]. Recent data given by Illingworth et al. [15] allowed us to investigate aspects of epigenetic regulation for their contribution to bimodal gene regulation. These authors surveyed methylation within blood, brain, muscle, and spleen and obtained lists of genes with methylated CpG islands in 5', intragenic, and 3' regions, which mapped to roughly 6–8% of human genes. The genes identified with intragenic DNA methylation were more common among the set of bimodal genes, suggesting that the inhibitory effect of DNA methylation on transcription elongation [11] may be a regulatory mechanism for bimodal genes. We also used the methylation data from Illingworth et al. [15] to test the relationship between DNA methylation status and the mode of expression within bimodal genes. The results varied for each tissue type, with the largest differences being decreased DNA methylation in bimodal genes with a "high" mode of expression in brain and increased DNA methylation in bimodal genes with a "high" mode of expression in muscle. DNA methylation is typically considered a gene silencing mechanism, which would correspond to low expression. However, the very small portion of genes represented in the CpG island methylation data for these four tissues may not be an adequate set to observe a consistent trend.

### Histone methylation provides a switching mechanism for bimodal genes

**Figure 4:**
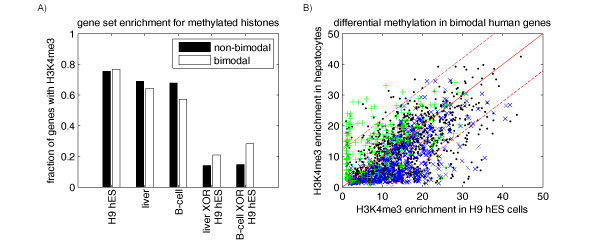
**Bimodal gene enrichment for promoter region methylation of lysine 4 of histone H3 (H3K4me3)**. A) Fraction of bimodal vs. non-bimodal genes enriched for histone methylation within their promoters as reported by Guenther et al. [12] for H9 hES cells, liver (hepatocytes), and B-cells. The fourth and fifth sets of bars represent the set of genes enriched at high confidence within one tissue but not the other liver versus hES cells and B-cell versus hES cells. B) H3K4me3 enrichment ratio from Guenther et al. [12] for liver vs. stem cells is shown for bimodal genes. Genes expressed with "high" mode in liver and "low" mode in H9 stem cells are shown with green "+" symbols, while bimodal genes expressed with "low" mode in liver and "high" mode in stem cells are shown with blue "x" symbols. Black points are used for the remaining bimodal genes expressed in common modes between these two tissue types. The standard deviation around the line y = x (solid red line) is shown as dashed red lines.

Next, we considered the possible role of epigenetic regulation as a switching mechanism for bimodal genes. A recent dataset that mapped histone modifications across the human genome for three cell types, including human embryonic H9 stem cells (hES), liver cells (hepatocytes), and B-cell lymphocytes [12] was used to evaluate the enrichment of histone 3 lysine 4 trimethylation (H3K4me3) at the promoters of bimodal genes. The H3K4me3 enrichment based on each of these three tissue types did not suggest a role in the regulation of bimodal genes (Figure 4A). However, if histone methylation provided a switching mechanism for bimodal gene expression, this would be evident in the differential methylation between tissue types, and not methylation status pertaining to a single tissue type. We used the data from these three tissues to create lists of genes with differentially enriched H3K4me3 regions for liver versus H9 hES cells and for B-cells versus H9 hES cells. These sets of differentially enriched H3K4me3 regions appeared with 50 to 100% higher frequency in bimodal genes compared to non-bimodal genes, as seen in Figure 4A. To further investigate the correlation between histone methylation and bimodal gene expression, we gathered additional microarray samples corresponding to H9 stem cells (GEO dataset accession numbers GSE9865, GSE8884, and GSE2248) and evaluated the mode of expression for bimodal genes within those H9 stem cell samples as well as liver samples within our dataset. We identified a group of bimodal genes as I) "high" in liver but "low" in stem cells, II) "low" in liver but "high" in stem cells, and III) expressed in common modes between these two tissues ("high" in both or "low" in both). These results are plotted in Figure 4B. Group I (green "+" symbols in Figure 4B) had a corresponding increase in methylation enrichment for liver vs. stem cells for nearly 85% of the genes, while group I (blue "x" symbols in Figure 4B) had a corresponding decrease in liver vs. stem cells for 77% of the genes. Approximately, 65% of the remaining bimodal genes expressed in common modes between these two tissue types (black points in Figure 4B) were within the standard deviation around the line y = x. These results demonstrate a strong association between histone methylation status and the mode of expression for bimodal genes.

## Discussion

In this study, using a large-scale microarray database, we have annotated 1847 human genes as having bimodal gene expression profiles. A recent study used again a large human microarray dataset for cancer samples to identify nearly 800 bimodal genes with the employment of a model-based clustering algorithm [7]. A comparison of their list against our list of bimodal genes resulted in 285 common elements, suggesting that bimodal genes in our list may not perform as bimodal in disease states in addition to possible switching of expression state in a disease state from one mode to another. Even in healthy tissue comparison, orthology argument did not entirely preserve bimodality in mouse and human data. Nearly 40% of the genes in this list corresponded to human orthologs of mouse bimodal genes that were annotated in our previous study [1]. Bimodality within 40% of human-mouse orthologs can be viewed as substantial overlap when considering that besides measurement noise and slightly different tissue types represented by datasets for each organism, differences exist in transcript sequences and transcript regions targeted by the microarray probes for orthologous genes among the two species. Further differences in gene expression between the two species arise from changes in regulatory sequences resulting from evolution [5,6]. This overlap demonstrates some degree of stability of bimodality in these datasets, even though we did not force identical tissue type quantities between the two organisms.

Our study shows that bimodal genes make a large contribution to the proteins composing the extracellular matrix as well as external membrane proteins. The enrichment within GO cellular component terms may at first appear contradictory, since the results include disparate terms related to the plasma membrane, cytoplasm, and extracellular matrix, while the KEGG pathway findings more highlighted extracellular communication. However, GO terms do not have a direct correspondence with KEGG pathways and gene membership is not exclusive to a single term. Mapping KEGG pathways to GO cellular component reveals that 66% of the bimodal genes in Focal Adhesion are contained in the cytoplasm; 40% of genes in both ECM-receptor interaction and Focal adhesion are contained in the GO cellular component "plasma membrane." Within the cell membrane side of the ECM-receptor interaction pathway, integrin subunits α7, β1, and β6 were identified as bimodal, while several others, including α2–α6 and β3–β5 were not. This finding suggests that integrin complexes are regulated by an interplay of transcript-level regulation as well as previously shown post-translational modifications [16,17]. In addition, several bimodal genes in the focal adhesion pathway are linked to phosporylation of β-catenin, a key element in the Wnt – signaling pathway, which plays a functional role in cell fate, proliferation, and apoptosis [18]. The Wnt-signaling pathway is active in development and is also a culprit in disease such as colorectal cancer and melanomas [18,19]. As such, bimodal genes upstream from these interactions provide potential markers for tissue-specific signaling as well as metabolic and chronic diseases.

Functional enrichment analysis of "high" and "low" expression subsets of the bimodal genes reveals that they play a role of activation and deactivation of tissue-specific pathways (table 4) and processes (Table 5). Bimodal gene sets involved in the Focal adhesion and ECM-receptor pathways demonstrated consistent modes of expression across human and mouse for brain, heart, and skeletal muscle, the three tissue types with the largest amount of samples in our investigation (Table 4). GO enrichment for "high" and "low" mode gene sets showed consistency between tissue-specific modes of expression in human and mouse, demonstrated by biological processes such as synaptic transmission in brain, and muscle contraction in skeletal muscle (Table 5). The consistency in expression modes of bimodal genes in the mouse and human is further reinforced by brain-specific expression for the cellular components of synapse, post-synaptic membrane, and muscle-specific expression for structural components of muscle, such as sarcoplasmic reticulum, collagen, and cytoskeleton (Table 5). Taken together, these findings indicate that the mode of expression for bimodal genes plays a role in the stable differentiation of specialized tissues, and pathway-specific usage of these genes is conserved across human and mouse in several cases.

Bimodality implies a high degree of transcript-level regulation, and bimodal genes may act as switches for the direction of signals and/or metabolic flow. Our study shows that bimodality appears to arise independently from the type of promoter present, even though we estimate the number of TATA boxes in bimodal genes is enriched, appearing in over 80% of bimodal genes with documented promoter sites. This may merely reflect a bias in gene annotation, as the involvement of these genes among pathways of interest, such as MAPK signaling and ECM-receptor interaction, may draw more focus for experimentation. The number of alternative (promoter) transcription start sites appears to have an influence on the bimodality of gene expression. Unlike the limited number of experimentally produced promoter binding sites, alternative promoter sites have been assessed by genome-wide mapping of transcript 5' ends [14]. While the number of known alternative promoter start sites for bimodal genes is shifted to a higher number than for non-bimodal genes, it is not sufficient to explain the phenomenon of bimodality. Additionally, previous studies investigating the usage of alternative promoters by gene ontology cellular component reveal that genes with several alternative promoters play a role in signaling, but do not contribute to the extracellular region, suggesting a difference from the set of bimodal genes [20]. This still allows for bimodal genes to include a subset of genes with a higher than average number of alternative promoters that work in concert with other regulatory mechanisms such as DNA methylation and histone modification.

We have shown that bimodal gene expression has a bias for multiple alternative promoters, as well as an association with histone methylation (H3K4me3), though a complete description of the links between all possible regulatory mechanisms cannot be made with currently available data. A recent study has shown that CpG-specific RNA polymerase II binding, associated with transcription initiation, is conserved among different tissue types [21]. A large portion of these may constitute the set of housekeeping genes, while others may appear at high modes of expression in some tissues, while silenced in other tissues via CpG methylation. A link has also been demonstrated between DNA methylation and histone methylation, where genes that undergo transcription initiation require H3K4 methylation as well as unmethylated DNA [22,23]. Consistent with our findings in this study, H3K4 trimethylation was previously associated with transcriptionally active genes [24]. The presence or absence of this modification can achieve switch-like regulation [25,26]. Histone methylation, along with DNA methylation, is a key player in cell differentiation during development and maintain cell lineage [15,27]. This stable regulation also maintains the balance between cell communication molecules and the extracellular environment [28]. Aberrant histone methylation patterns are among the epigenetic modifications that give rise to cancer [29]. As the mode of expression for bimodal genes is closely related to H3K4me3 status, gene expression levels may be used as a surrogate for detecting aberrant patterns of methylation associated with disease. While our knowledge of methylation associated with gene regulation may be incomplete, genes regulated through alternative promoters have an additional layer of complexity, as they can have largely different methylation status at individual promoters from tissue to tissue [30]. The regulatory mechanism for bimodal genes may therefore include a complex logic of DNA and histone methylation among alternative promoters, in addition to positive and negative regulation through transcription factor binding.

Alternative splicing events may present another explanation for bimodality in the expression of genes. Alternative-splicing isoforms have been identified as tissue-specific [31]. A substantial portion of alternative splicing isoforms are also associated with nonsense-mediated mRNA decay [32]. Three out of five genes identified with muscle-specific alternative splicing in Xu et al. [31] (PDLIM7, TPM2, and FHL1) were identified as bimodal and expressed in "high" mode in our microarray data for muscle but not other tissues. Five out of the twenty-two genes identified as having brain-specific alternative splicing in Xu et al. [31], were identified as having bimodal expression, but only one of these (CDC42) was expressed in a brain-specific manner. This indicates the possibility that stable transcript splice isoforms account for the high mode of expression in specific tissues, while alternative splice isoforms undergo nonsense-mediated decay. The methylation/promoter analysis presented here is a first step towards understanding the complex interplay of various molecular mechanisms affecting transcription in human and mouse genomes.

## Conclusion

This research expanded our representation of "switch-like" gene expression by cataloging the bimodal genes evident in human microarray data for diverse tissue types. Results obtained from human data affirm that genes with bimodal, switch-like expression play a large role in cells communication with the extracellular environment. Tissue-specific modes of expression among the bimodal genes organized by KEGG pathways and GO cellular component revealed that they play a role in tissue specialization that is in common between human and mouse. Equally as important, our results indicate bimodal genes capture epigenetic aspects of gene regulation, indicative of gene expression levels that are stable across cell divisions. These findings verify that biologically relevant information can be inferred from bimodal distributions, much in the way that housekeeping genes have been used. Because the "high" mode of expression modes corresponds well with histone methylation enrichment in promoter regions, bimodal genes may serve as biomarkers for complex diseases such as cancer, where aberrant histone methylation is a known factor in disease progression. Through the identification of condition-specific modes of expression within healthy tissue and disease subtypes, the method presented allows for an alternative approach to differential gene expression analysis.

## Methods

### Data Selection

Human gene expression microarray datasets were obtained from both the Gene Expression Omnibus (GEO) [33,34] and ArrayExpress [35,36] online repositories. For the purpose of comparing a subset of human bimodal genes with those identified in mouse, we created a microarray dataset with comparable tissue samples to those used in the mouse study (Table 1) [1]. In order to adequately represent some tissue types, it was necessary to combine datasets from Affymetrix HGU133A and HGU133plus2 arrays, which have 22,277 probesets in common.

### Microarray normalization

Affymetrix probe intensities were filtered to exclude probesets that are not shared between the HGU133A and HG133plus2 microarrays. The remaining probesets were normalized using Robust Multichip Average (RMA) background correction, quantile normalization, and summarization approach for large datasets described as the refRMA algorithm [37]. RMA background adjustment was performed on each chip. Quantile normalization was performed by computing probe-level quantiles from a 940-array (HGU133plus2) training set (Additional File 1) and applying these quantiles to additional HGU133A arrays as they were added to the dataset. RMA summarization was performed by median polishing on the 940-array training set and storing the row effects (probe effects) to be applied to additional arrays as they were added to the dataset. The quantile normalization and row effect vectors are provided in Additional File 1.

### Gene annotation

Annotation for Entrez Gene ID, EMBL accession, gene symbol, and gene ontology biological process, cellular component, and molecular function were retrieved from the HGU133plus2 and MGU74Av2 annotation files updated March 2008 on the Affymetrix website [38]. KEGG pathway descriptions were retrieved April 29th, 2008 from the KEGG ftp site [3,39]. Orthologous gene pairs between mouse and human were identified using the EMBL accession number database from OMA browser [40,41], dated November 2007, in addition to matching official gene symbols. The data were then imported to Matlab R2007b (The Mathworks Inc., Natick, MA, USA), where all subsequent procedures were implemented.

### Identification of bimodal genes in the human genome

**Figure 5:**
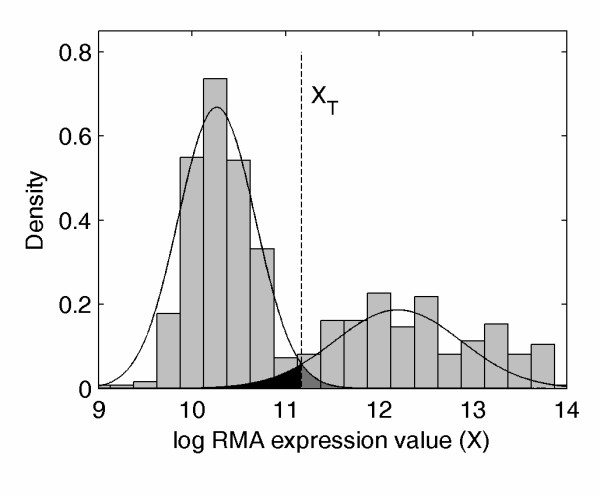
**Bimodal gene expression**. The histogram and normal mixture probability density function (pdf) are shown for a typical bimodal gene. The threshold between the high and low mode of expression is labeled as X_T _and darker shading is used to represent the misclassification region.

Bimodal genes were identified in the human genome by fitting a two-component mixture model, as detailed in the methods of our previous work [1]. Briefly, we tested the hypothesis H_1 _that gene expression distribution follows a two-component (bimodal) mixture against the hypothesis H_0 _of a single normal distribution, adjusted for skewness using a box-cox transformation. The log likelihood ratio test statistic -2logλ was computed for the two-component mixture hypothesis H_1 _versus the null hypothesis H_0 _of a single component. Candidates for bimodal "switch-like" genes were selected as those with p-values no more than 0.001 based on a chi-square distribution with six degrees of freedom at the values of -2logλ. This subset of genes was further reduced by the imposing the requirement that the standardized area of intersection A was less than 0.10. We define A as the area of overlap between the two components, representing type I and type II error for the estimated bimodal distribution, divided by the total area. A typical two-component representation of bimodal gene expression is depicted in Figure 5. To verify that chip effect differences between the HGU133plus2 and HGU133A did not influence the identification of bimodal genes, this procedure was applied to the subset of samples both including and excluding HGU133A arrays. Muscle tissue was selected for this due to the large number of samples and the roughly equal portions of HGU133plus2 and HGU133A arrays for this tissue type. Bimodal expression patterns were identified within muscle tissue first for the HGU133plus2 samples, then for all muscle samples, which adds in potential chip effects as well as lab effects. Identification of bimodal profiles within these samples demonstrated consistency across array types, as classification was consistent for 97% of probesets.

### Functional enrichment analysis

KEGG pathway and GO annotations described above were used to compute functional enrichment scores for all switch-like genes. Functional enrichment analysis was performed in Matlab by calculating the ratio of genes belonging to a functional category within a gene set of interest against the total number of genes belonging to that functional category within the 22,277 common probesets between the HGU133A and HGU133plus2 arrays. Enrichment p-values were computed from a hypergeometric distribution [42]. The p-value cutoffs were selected at 0.01 for KEGG pathways and 0.001 for GO terms, to reduce the false discovery rate. The set of candidate bimodal genes was distributed among 186 unique KEGG pathways and 618 unique GO cellular component terms, for which an expected 1.9 and 0.6 of the terms may appear significant by chance at these p-value cutoffs, respectively. Functional enrichment analysis was repeated within the list of significant terms to identify tissue-specific behavior within brain, heart, and muscle tissue. These three tissues were selected because they are represented by a large number of samples within the data, and several terms that should be specific to these tissue types were identified as significantly enriched. Using the binomial distribution approach detailed in [1], we identified bimodal genes that were expressed either "high" or "low" within these three tissue types. KEGG pathway and GO subsets were tested for enrichment for sets of "high" and "low" mode genes against the hypergeometric distribution with p = 0.05.

### Promoter analysis

The set of bimodal genes was evaluated for regulatory mechanisms including the core promoter type and the number of alternative promoters. Genes were separated into subsets of bimodal and non-bimodal for promoter analysis to evaluate differences within each of human and mouse. Promoter sites and sequence motifs were obtained from MPromDB [13]. Promoters corresponding to the targets of transcription factors were mapped to the sets of bimodal and non-bimodal genes using Entrez gene ID. The remaining promoter types seldom appeared and were bundled together into an "other" category. The frequency that each of these regulatory sites, including AP-1, AP-2, SP1, TATA and CAAT-signal, appear within bimodal genes and non-bimodal genes was assessed for the bimodal and non-bimodal subsets in human and mouse. Additional annotation for human and murine genes describing the number of alternative promoter sites (hspromoter.tab for human and mmpromoter.tab for mouse, both dated June 12th, 2007) were downloaded from the database of transcriptional start sites (DBTSS) [14]. The distribution of alternative promoters was computed as a histogram within the sets of bimodal genes and non-bimodal genes within human and mouse. The statistical significance for two, three, four, and two or more promoters was estimated using the hypergeometric distribution.

### Analysis of DNA methylation effect on mode of expression

DNA methyaltion was explored as a regulatory mechanism for bimodal genes due to its known association with gene silencing. CpG methylation data were obtained from Illingworth at al.; supplementary information Dataset S1 [15]. This dataset documents methylation sites for roughly 8% of the human genome across four tissues – blood, brain, muscle, and spleen. The frequency of methylation sites that were mapped to 5', 3', and intragenic regions of known genes was computed and significance of enrichment within bimodal genes was estimated from the hypergeometric distribution. This methylation data were also used to evaluate correspondence between methylation status within either the 5' or intragenic regions and the mode of expression in bimodal genes. Genes were assigned to a mode of expression within each of the four tissues by treating expression measurements within each tissue as Bernoulli trials against the binomial distribution, as described in [1]. The frequency of DNA methylation was then calculated in the subsets of "high" and "low" genes for each of the four tissues.

### Comparison of histone methylation enrichment versus mode of expression

The final regulatory mechanism that was assessed for a contribution to bimodal gene expression was histone methylation. Methylation data were obtained from Guenther et al. Table S3 and Table S4, which describes H3K4me3 enrichement scores and locations across the human genome for three cell types: human embryonic stem cells (hES), liver cells (hepatocytes), and B-cell lymphocytes [12]. Enrichment scores for H3K4me3 designated as high-confidence in Guenther et al. were used to create a gene set for each of these three tissue types [12]. Two additional gene sets were created based on differential H3K4me3 enrichment for liver versus H9 hES cells and for B-cells versus H9 hES cells. For example, the liver versus H9 hES gene set includes those enriched with high confidence in liver but not H9 hES cells in addition to those with high confidence in H9 hES cells but not liver. The frequency of histone methylation sites based on these three tissues as well as the differentially enriched sites were evaluated for the sets of bimodal genes and non-bimodal genes within human. Additionally, the significance of each list of sites was evaluated using the hypergeometric distribution.

To further investigate the interplay between histone methylation and bimodal gene expression, we gathered additional microarray samples corresponding to H9 stem cells (samples GSM249282, GSM225045, and GSM38629, from datasets GSE9865, GSE8884, and GSE2248, respectively) and evaluated the mode of expression for bimodal genes within that those H9 stem cells as well as liver samples within our dataset. Using the binomial distribution approach detailed in [1], we identified a group of bimodal genes as I) "high" in liver but "low" in stem cells, II) "low" in liver but "high" in stem cells, and III) expressed in common modes between these two tissues ("high" in both or "low" in both). These three subsets were then used to create a scatter plot of H3K4me3 enrichment, excluding genes with enrichment scores below a 2-fold enrichment in both hES and hepatocytes.

## Authors' contributions

AE and AT worked together on this project. AE implemented the algorithms, performed the computations and provided a first draft for the manuscript. Both authors read and approved the final version of the manuscript.

## Supplementary Material

Additional file 1 **Microarray samples used for the identification of human bimodal genes.** This file contains a worksheet "samples used" listing the HGU133A and HGU133plus2 samples used for identification of bimodal genes, a worksheet "HGU133plus2 training set" listing all HGU133plus2 samples used for RMA quantile normalization and summarization, a worksheet "array quantiles" listing array quantiles, and a worksheet "row effects" listing row effects for 22,277 probesets calculated during the RMA summarization step. . Click here for file

Additional file 2 **Bimodal genes identified in the human genome.** This table provides a comprehensive list of bimodal genes for the human genome including parameters used in the identification of bimodal gene expression. . Click here for file

Additional file 3 **Bimodal genes within significant KEGG pathways.** This file provides a list of bimodal genes organized into KEGG pathways that were identified as significantly enriched. . Click here for file
